# An Uncharacterized Major Facilitator Superfamily Transporter From *Planococcus maritimus* Exhibits Dual Functions as a Na^+^(Li^+^, K^+^)/H^+^ Antiporter and a Multidrug Efflux Pump

**DOI:** 10.3389/fmicb.2018.01601

**Published:** 2018-07-16

**Authors:** Heba Abdel-Motaal, Lin Meng, Zhenglai Zhang, Amro H. Abdelazez, Li Shao, Tong Xu, Fankui Meng, Shaima Abozaed, Rui Zhang, Juquan Jiang

**Affiliations:** ^1^Key Laboratory of Agricultural Microbiology of Heilongjiang Province, and Department of Microbiology and Biotechnology, College of Life Sciences, Northeast Agricultural University, Harbin, China; ^2^Department of Microbiology, Agriculture Research Center, Soils, Water, Environment and Microbiology Research Institute, Giza, Egypt

**Keywords:** slight halophile, *Planococcus maritimus*, major facilitator superfamily, Na^+^(Li^+^, K^+^)/H^+^ antiporter, multidrug efflux pump

## Abstract

Within major facilitator superfamily (MFS), up to 27 unknown major facilitator families and many members of 60 well-characterized families have been functionally unknown as yet, due to their sharing no or significantly low sequence identity with characterized MFS members. Here we present the first report on the characterization of one functionally unknown MFS transporter designated MdrP with the accession version No. ANU18183.1 from the slight halophile *Planococcus maritimus* DS 17275^T^. During the screening of Na^+^/H^+^ antiporter genes, we found at first that MdrP exhibits Na^+^(Li^+^, K^+^)/H^+^ antiport activity, and propose that it should represent a novel class of Na^+^(Li^+^, K^+^)/H^+^ antiporters. However, we speculate that MdrP may possess an additional protein function. The existence of the signature Motif A of drug/H^+^antiporter (DHA) family members and phylogenetic analysis suggest that MdrP may also function as a drug efflux pump, which was established by minimum inhibitory concentration tests and drug efflux activity assays. Taken together, this novel MFS transporter exhibits dual functions as a Na^+^(Li^+^, K^+^)/H^+^ antiporter and a multidrug efflux pump, which will be very helpful to not only positively contribute to the function prediction of uncharacterized MFS members especially DHA1 family ones, but also broaden the knowledge of Na^+^/H^+^ antiporters.

## Introduction

Major facilitator superfamily (MFS) is the largest and most diverse known superfamily of secondary transporters widely distributed throughout the whole living world (Pao et al., 1998; Law et al., 2008; Reddy et al., 2012), which has been currently divided into 87 recognized families with more than one million sequenced members on the basis of phylogenetic analysis in the Transporter Classification Database (TCDB) (Saier et al., 2016). Mechanistically, MFS transporters have been summarized to include three distinct categories: (i) uniporters only transporting one type of substrate driven by the substrate gradient, (ii) symporters simultaneously translocating two or more substrates in the same direction by using the electrochemical gradients, and (iii) antiporters transporting two or more substrates in the opposite direction across the membrane (Law et al., 2008). For topological and sequential characteristics, most MFS transporters consist of either 12, 14, or occasionally, 24 transmembrane segments (TMSs) containing 400 to 600 amino acid residues (Pao et al., 1998; Law et al., 2008; Reddy et al., 2012). Up to now, 60 characterized families within this superfamily have been reported to transport a diverse range of substrates such as sugars, amino acids, vitamins, drugs, organic and inorganic anions and cations, and etc. (Law et al., 2008; Reddy et al., 2012). However, the additional 27 recognized families have been functionally uncharacterized as yet and designated unknown major facilitator (UMF) families (Saier et al., 2016). Also, each well-characterized family within MFS includes many members derived from the genome sequencing projects, whose functions have been unascertained as yet due to their significantly low sequence identity with the characterized members (Saier et al., 2016).

Among 60 characterized families within MFS, single-drug or multi-drug resistance proteins have been categorized into four major drug/H^+^antiporter (DHA1-4) families based on the number of TMSs and the variation of the signature motif designated Motif A between TMS2 and TMS3 of DHA family drug efflux pumps (Saier et al., 2016). DHA1 (previously designated DHA12 due to their containing 12 TMSs) is the largest one of four DHA families consisting of 107 members in TCDB (TC No. 2.A.1.2.1 to 2.A.1.2.107), which contain 12 TMSs and the signature Motif A with the consensus sequence of GxLaDrxGrkxxl (x standing for any amino acid; capital and lowercase letters representing amino acid frequency of >70% and 40–70%, respectively) (Henderson and Maiden, 1987; Griffith et al., 1992; Paulsen et al., 1996). DHA2 (previously designated DHA14 due to their containing 14 TMSs) is another one larger DHA family including 77 members in TCDB (TC No. 2.A.1.3.1 to 2.A.1.3.77). Although it contains the same Motif A as DHA1, the sole difference is that DHA2 contain 14 TMSs. In comparison with the former two DHA families, DHA3 is one relatively smaller DHA family including 24 members in TCDB (TC No. 2.A.1.21.1 to 2.A.1.21.24), which contain 12 TMSs and a variant of Motif A with the consensus sequence of E-x-P-x-x-x-x-x-D-x-x-x-R-K (Bannam et al., 2004). DHA4 is a recently-recognized DHA family including one characterized BC3310 (previously belonging to UMF-2 family) from *Bacillus cereus* and two uncharacterized members (TC No. 2.A.1.26.1 to 2.A.1.26.3), which contain 12 TMSs and a variant of Motif A with the consensus sequence of E-r/k-P-L-x-r/k-x-G-x-r/k-P-x-I (Kroeger et al., 2015). Many DHA members have been well characterized in the pathogenic and non-pathogenic bacteria (Bolhuis et al., 1995; Paulsen et al., 1996; Nishino and Yamaguchi, 2001; Kumar and Varela, 2012; Kumar et al., 2013; Kroeger et al., 2015). However, a considerable number of uncharacterized members from the sequenced microbial genomes have been increasingly classified into these four families especially DHA1 and DHA2 on the basis of the variation of Motif A, phylogenetic relationship and the number of TMSs, although they share low identities with the well-characterized members (Saier et al., 2016). In addition to the above-mentioned DHA1-4 families, *Escherichia coli* Fsr of Fsr family and *Stenotrophomonas maltophilia* TrcA of MocC family within MFS have been also identified to be bacterial multiple drug pumps (Nishino and Yamaguchi, 2001; Chang et al., 2011). Apart from MFS, drug efflux pumps have been also classified into other four major families/superfamilies including ATP-binding cassette (ABC) superfamily (Lubelski et al., 2007), resistance-nodulation-division (RND) family (Tseng et al., 1999), small multidrug resistance (SMR) protein family (Bay et al., 2008), and multidrug and toxic compound extrusion (MATE) family (Kuroda and Tsuchiya, 2009), mainly based on their significant difference in sequence identity, substrate specificity, number of components (single or multiple), number of TMSs and energy source.

Strain DSM 17275^T^ was isolated from sea water of a tidal flat of the Yellow Sea in South Korea, and identified to represent a novel species of the genus *Planococcus, Planococcus maritimus* (Yoon et al., 2003). In our previous study on the identification of another novel species of the genus *Planococcus, P. dechangensis*, this strain was found to be a slight halophile with the growth range of NaCl concentrations of 0.17–2.91 M (optimum, 0.34 M) (Wang et al., 2015). As the type strain of the species *P. maritimus*, the genome of strain DSM 17275^T^ with the total length of 3.28072 Mbp has been also sequenced and recently released. Among 3, 107 predicted proteins, many proteins including 28 MFS transporters have been non-annotated as yet, maybe due to their low identity with experimentally characterized proteins. We speculate it is very likely that this strain, which can tolerate up to 2.91 M NaCl, contains a variety of important Na^+^/H^+^ antiporters, since almost all halophilic microorganisms have the ability to expel Na^+^ from the interior of the cells using Na^+^/H^+^ antiporters (Ventosa et al., 1998; Oren, 1999). Also, we expect to obtain even non-annotated proteins with the capability of displaying Na^+^/H^+^ antiport activity from this strain, since its genome sequence has revealed the existence of a considerable number of non-annotated proteins.

During the screening of Na^+^/H^+^ antiporter genes, we found as expected that one uncharacterized MFS transporter designated MdrP from strain DSM 17275^T^ functions as a Na^+^(Li^+^, K^+^)/H^+^ antiporter. However, we found that MdrP shares no identity with three MFS members reported to exhibit Na^+^/H^+^ or Na^+^(K^+^)/H^+^ antiport activity, including DHA1 family multidrug efflux pumps, MdfA (Edgar and Bibi, 1997) and MdtM (Holdsworth and Law, 2013), and DHA2 family tetracycline/H^+^ antiporter, Tet(L) (Cheng et al., 1994). Enlightened by these three reports, we speculate that MdrP may possess an additional protein function such as drug efflux pump or etc., besides Na^+^(Li^+^, K^+^)/H^+^ antiport activity. Although it only shares a quite low identity with the solely two characterized DHA1 family multidrug efflux pumps, *Lactococcus lactis* LmrP (Bolhuis et al., 1995) and *E. coli* MdtH (Nishino and Yamaguchi, 2001), phylogenetic analysis showed that MdrP can constitute a stable separate cluster with LmrP and MdtH with a bootstrap value of 89%. Therefore, we attempted to identify whether MdrP can also function as a multidrug efflux pump like LmrP, MdtH or both. Finally, we found and reported here that this novel MFS transporter, MdrP, exhibits dual functions as a Na^+^(Li^+^, K^+^)/H^+^ antiporter and a multidrug efflux pump.

## Materials and Methods

### Strains and Growth Conditions

All the strains related to this study were presented in Supplementary Table S1. *P. maritimus* DSM 17275^T^ was incubated in modified Luria-Bertani (LB) medium with the composition of 1.0% tryptone, 0.5% yeast extract plus 0.34 M NaCl (optimal) at pH 7.2–7.4, 28°C (Wang et al., 2015). *E. coli* KNabc (Nozaki et al., 1996) with the absence of three major Na^+^/H^+^ antiporters (NhaA, NhaB, and ChaA) and its transformants were grown overnight to OD_600_ of 1.0 at 37°C in KCl-modified LB (LBK) medium with the substitution of NaCl by 87 mM KCl, as described by our recent studies (Dong et al., 2017; Meng et al., 2017; Wang et al., 2017). Growth tests for salt tolerance or alkaline pH resistance were performed by growing *E. coli* KNabc transformants in LBK media with the addition of NaCl or LiCl at indicated concentrations or supplemented by 50 NaCl with the adjustment of pH by Tris-HCl buffer at the final concentration of 100 mM, as described by our recent studies (Dong et al., 2017; Meng et al., 2017; Wang et al., 2017). Na^+^/H^+^ antiporters extrude Na^+^ or Li^+^ to the exterior of cells by the influx of H^+^ (Krulwich et al., 2011; Quinn et al., 2012; Meng et al., 2014; Padan, 2014). Therefore, the addition of 50 mM NaCl was required for the test for alkaline pH resistance provided by antiporters of this category. The drug-sensitive *E. coli* mutant CM2 was constructed through the disruption of one major multidrug efflux system, AcrAB (Ma et al., 1995), of *E. coli* DH5α with the aid of pKD3 as a template of chloramphenicol resistance gene and pKD46 containing a aaa Red recombinase system (Supplementary Table S1), according to the protocol described by Datsenko and Wanner (2000). Ampicillin was used for the selection and growth of *E. coli* KNabc transformants at a final concentration of 50 μg/ml. Preparation and electroporation of *E. coli* electrocompetent cells were performed as described in our previous study (Jiang et al., 2013a).

### Screening of Na^+^/H^+^ Antiporter Gene

Genomic DNA from strain DSM 17275^T^ was partially digested by *Sau*3AI, followed by the separation and purification of 0–10 kb DNA fragments by agarose electrophoresis. Also, *Bam*HI-digested pUC18 was dephosphorylated by a bacterial alkaline phosphatase. The ligation mixture of purified DNA fragments with treated pUC18 by a T4 DNA ligase was then electroporated into *E. coli* KNabc as described in our previous study (Jiang et al., 2013a). Positive clones were obtained by functional complementation on LBK medium plates containing 0.2 M NaCl. The subcloning of each ORF was carried out for the establishment of Na^+^/H^+^ antiporter gene through its fusion in frame with an N-terminal His_6_ tag into an expression vector pET19b (Novagen Ltd., United States). The resultant construct was verified by sequencing for its accuracy. All the plasmids and primers related to this study were presented in Supplementary Table S1.

### Preparation of Everted Membrane Vesicles

Everted membrane vesicles were prepared from *E. coli* KNabc/pET-mdrP and KNabc/pET19b (as a negative control) according to the protocol described by Rosen (1986). Cells were collected at the mid-exponential phase by centrifugation at 5000 *g*, 4°C for 10 min and re-suspended in the 10 mM Tris-HCl (pH 7.5) buffer containing 140 mM choline chloride, 0.5 mM dithiothreitol, 250 mM sucrose, a tablet of protease inhibitor (Roche) and 1 mM phenylmethylsulfonyl fluoride. Cell suspension was broken at 2000 psi via a JG-1A French pressure cell press (NingBo Scientz Biotechnology Co., Ltd, China), followed by centrifugation at 10,000 *g*, 4°C for 10 min. Partial supernatant was sampled as cell extract and the remaining one continued to go through 1-h ultracentrifugation at 100,000 *g* for the separation of membrane fraction (pellets) from cytoplasmic fraction (supernatant). It should be stressed that membrane fraction exists as everted membrane vesicles using the above-mentioned protocol. Partial cytoplasmic fraction was sampled and everted membrane vesicles were re-suspended in the same buffer as above and then stored at -80°C. Protein concentration was determined by using bovine serum albumin as a standard according to the method by (Lowry et al., 1951).

### Detection and Localization of MdrP by Western Blot

Detection and localization of MdrP were carried out by using the above-mentioned samples for cell extract, cytoplasmic fraction and membrane fraction from *E. coli* KNabc/pET-mdrP and KNabc/pET19b. SDS-PAGE and western blots were performed as described by our recent studies (Dong et al., 2017; Meng et al., 2017). A rabbit anti-His_6_ tag antibody (Abcam Ltd., China) was employed for His_6_-tag detection, together with a goat anti-rabbit secondary antibody conjugated with horseradish peroxidase (Nachuan Biotechnology Co., Ltd., Changchun, China). Antibody binding was visualized on the basis of chemiluminescence immunoassay by using a BeyoECL Star kit (Beyotime Biotechnology Co., Ltd., China) via a Tanon-5200 multi chemiluminescence imaging system (Tanon Co., Ltd., China).

### Na^+^(Li^+^, K^+^)/H^+^ Antiport Assays

Na^+^(Li^+^, K^+^)/H^+^ antiport activity was determined at indicated pH values by using everted membrane vesicles through the acridine orange fluorescence dequenching method as described by our recent studies (Dong et al., 2017; Meng et al., 2017; Wang et al., 2017). The vesicles (equivalent of 40 μg total membrane protein) were re-suspended in the assay mixture containing 140 mM choline chloride, 5 mM Mg_2_SO_4_, 2 μM acridine orange at the indicated pH values from 6.5 to 9.5 adjusted by a 10 mM BTP (Bis-TrisPropane) buffer. The fluorescence quenching with the acridine orange as a pH indicator was initiated by the addition of Tris-D-lactic acid at the final concentration of 5 mM, due to the respiration-coupled proton translocation from the outside of the vesicles into the inside. When the fluorescence quenching reached the steady state, a respiration-dependent proton gradient across the vesicles was constructed. NaCl, LiCl, or Na-free KCl with high purity (99.9995%, Sigma-Aldrich Co. LLC.) (To avoid the contamination of traces of NaCl) was added to the final concentration of 5 mM, the fluorescence could be dequenched on the basis of Na^+^, Li^+^ or K^+^ influx into the vesicles in exchange for proton efflux. The ratio of fluorescence dequenching extent by NaCl, LiCl, or KCl to the fluorescence quenching one by Tris-D-lactic acid was recorded as a respective representative of Na^+^(Li^+^, K^+^)/H^+^ antiport activity. Fluorescence was excitated at 492 nm (10-mm slit) and emission monitored at 526 nm (10-mm slit) via a Hitachi F-7000 fluorescence spectrophotometer (Hitachi Ltd., Tokyo, Japan).

### Calculation of *K*_0.5_ Values of MdrP for the Cations

The optimal antiport activity was determined at pH 9.0 for Na^+^(Li^+^)/H^+^ and at pH 8.5 for K^+^/H^+^ at the varied cation concentrations from 0 to 10 mM. *K*_0.5_ values of MdrP for the tested cations was calculated on the basis of non-linear regression analysis with the software Prism 5.0 by the plotting of the antiport activity as the respective functions of the corresponding cation concentrations, as described by our recent studies (Dong et al., 2017; Wang et al., 2017).

### Minimal Inhibition Concentrations (MICs) Tests

Minimal inhibition concentrations tests for *E. coli* CM2 and its transformants were carried out by using the twofold dilution method of drugs described by Kroeger et al. (2015). 1% overnight cultures of *E. coli* CM2 and its transformants were inoculated to 5-ml new LB medium and grown to an OD_600_ of 1.0 at 37°C, followed by the dilution of these pre-cultures to a final OD_600_ of 0.02. The test was carried out three times in triplicate in liquid LB medium with the addition of antimicrobial drugs in a twofold serial dilution. The cultures continued to be incubated at 37°C for 24 h and visually inspected for growth. The lowest concentration, at which the growth was inhibited completely, was recorded as the MIC.

### Assay for Ethidium Efflux by Whole Cells

Ethidium efflux assay was carried out by using the whole cells of *E. coli* CM2 transformants carrying pET-mdrP and the empty vector pET19b (as a negative control) as described by Masaoka et al. (2000). Cells were grown to an OD_600_ of 1.0 at 37°C in liquid LB medium and washed twice with the M9 minimal medium, and re-suspended in the same medium with a final OD_600_ of 0.2. Carbonylcyanide m-chlorophenylhydrazone (CCCP) and ethidium bromide were added to cell suspensions at the respective final concentrations of 40 and 2.5 μM. Cell suspensions continued to be shaken for 1 h at 37°C for the depletion of cell energy and energy-starved cells were pre-loaded with ethidium bromide. Thereafter, cells were harvested and washed twice with the M9 minimal medium supplemented with ethidium bromide (2.5 μM, final concentration) and re-suspended in the same medium with a final OD_600_ of 0.1. Cell suspensions were pre-incubated at 37°C for 5 min, glucose was added to cell suspensions at a final concentration of 20 mM to re-energize cells and initiate ethidium efflux assay. Fluorescence was excitated at 500 nm (10-mm slit) and emission monitored at 580 nm (10-mm slit) via a Hitachi F-7000 fluorescence spectrophotometer (Hitachi Ltd., Tokyo, Japan).

### Assay for Norfloxacin Accumulation in Whole Cells

Norfloxacin accumulation was measured in the whole cells of *E. coli* CM2 transformants carrying pET-mdrP and the empty vector pET19b (as a negative control) as described by Morita et al. (1998). *E. coli* CM2 transformants were grown to an OD_600_ of 1.0 in liquid LB medium supplemented with 40 mM potassium lactate. Cells were harvested and washed with 0.1 M Tris-HCl buffer (pH 7.0) and re-suspended in the same buffer to an OD_600_ of 1.0. After incubation at 25°C for 5 min, norfloxacin was added at a final concentration of 100 μM to initiate the assay. Samples (1 ml for each) were taken in triplicate on the indicated time points and centrifuged at 12,000 *g* for 30 s at 4°C and washed once with the same buffer. Where indicated, CCCP was used at a final concentration of 100 μM for the disruption of transmembrane proton gradient. The pellet was re-suspended on the indicated time points in 1 ml of 100 mM glycine-HCl (pH 3.0). Cell suspensions were shaken vigorously for 1 h at room temperature and then centrifuged at 12,000 *g* for 10 min at room temperature. The supernatant was diluted twofold with the same buffer, and the fluorescence was excitated at 277 nm (10-mm slit) and emission monitored at 448 nm (10-mm slit) via a Hitachi F-7000 fluorescence spectrophotometer (Hitachi Ltd., Tokyo, Japan).

### Bioinformatic Analysis

DNA was sequenced by Beijing Genomics Institute (Beijing, China) and analyzed with the software DNAMAN 6.0 for the detection of open reading frame (ORF). DNA sequence was submitted for promoter prediction to the website http://www.fruitfly.org/seq_tools/promoter.html. Transmembrane segment prediction and hydropathy analysis were performed at the website http://www.tcdb.org/analyze.php (Saier et al., 2016). Phylogenetic tree was constructed via the software MEGA 5.0 using the neighbor-joining method with a bootstrap analysis on the clustering stability (1000 replications) (Saitou and Nei, 1987), on the basis of the aligned protein sequences by the ClustalX program (Thompson et al., 1997). In order to download the putative MFS homologs, protein sequence of MdrP was aligned using BlastP at the NCBI (National Center for Biotechnology Information) website https://blast.ncbi.nlm.nih.gov/Blast.cgi?PROGRAM=blastp&PAGE_TYPE=BlastSearch&LINK_LOC=blasthome (Altschul et al., 1990). In order to search the characterized MFS homologs collected in the Transporter Classification Database (TCDB), protein sequence of MdrP was aligned using Blast at the TCDB website http://www.tcdb.org/progs/blast.php (Saier et al., 2016).

## Results

### Cloning of Na^+^/H^+^ Antiporter Gene and Sequence Analysis

**FIGURE 1:**
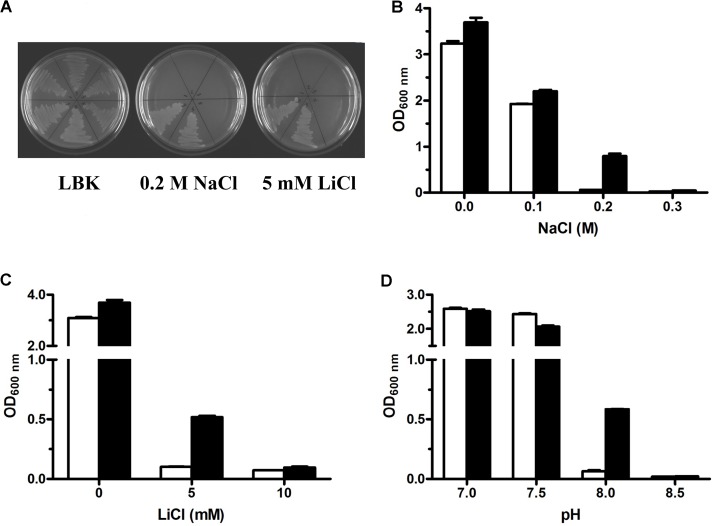
Growth tests for *Escherichia coli* KNabc and its transformants under saline or alkaline stress. For the complementation test **(A)**, *E. coli* KNabc and its transformants on LBK medium plates with no addition of NaCl or LiCl, 0.2 M NaCl or 5 mM LiCl. 1, KNabc only; 2, KNabc/pUC18; 3, KNabc/pET19b; 4, KNabc/pET-truncated uvrD; 5, KNabc/pUC-PM29; 6, KNabc/pET-mdrP. Detailed growth tests for salt tolerance or alkaline pH resistance were performed by growing *E. coli* KNabc transformants carrying pET19b (white column) and pET-MdrP (black column) in LBK media with the addition of NaCl **(B)** or LiCl **(C)** at indicated concentrations or supplemented by 50 mM NaCl with the adjustment of pH **(D)** by Tris-HCl buffer at the final concentration of 100 mM. Cell growth was ended after 24 h and monitored turbidimetrically at 600 nm. Each data point represents the average of three independent determinations.

Due to the deficiency in three major Na^+^/H^+^ antiporters (NhaA, NhaB, and ChaA), *E. coli* KNabc can’t grow in the presence of 0.2 M NaCl or 5 mM LiCl (Nozaki et al., 1996), either of which was routinely used as the selective stress condition for the cloning of Na^+^/H^+^ antiporter genes by functional complementation with *E. coli* KNabc. For the cloning of Na^+^/H^+^ antiporter gene, ligation mixture of genomic DNA fragments from *P. maritimus* DSM 17275^T^ with a cloning vector pUC18 was electroporated into *E. coli* KNabc, followed by the screening of its transformants on LBK medium plates containing 0.2 M NaCl. As a result, one recombinant plasmid designated pUC-PM29 containing one 3240-bp DNA fragment (Supplementary Figure S1) was found to succeed in complementing with *E. coli* KNabc (**Figure 1A**). Sequence analysis showed that this DNA fragment includes two ORF (ORF1 and ORF2), the sole latter of which is preceded by a predicted promoter and a Shine-Dalgarno (SD) sequence (Supplementary Figure S1). ORF1 beginning from No. 1 bp encodes a N-terminus truncated protein with no initiation codon, which corresponds with 100% identity to the partial amino acid sequence from No. 146 residue to No. 709 residue of one putative DNA helicase designated UvrD (accession version No. ANU18184.1) from *P. maritimus* DSM 17275^T^ (Supplementary Figure S1). ORF2 encodes an intact protein with an initiation codon and a stop codon, which corresponds with 100% identity to one uncharacterized MFS transporter (accession version No. ANU18183.1) from *P. maritimus* DSM 17275^T^ (Supplementary Figure S1). Because this uncharacterized MFS transporter was finally identified as a multidrug resistance protein, we designated it MdrP for the convenience of describing its identification as below. It should be emphasized that both 5′-end truncated *uvrD* gene and intact *mdrP* gene were inserted downstream of *lac* promoter in the opposite orientation in the recombinant plasmid pUC-PM29. Therefore, 5′-end truncated *uvrD* gene without the aid of any promoter or SD sequence is impossible to be transcribed or translated in *E. coli*. In contrast, *mdrP* together with its native promoter and SD sequence is the sole gene that can be active for functional complementation with *E. coli* KNabc.

### Alignment of MdrP With Its Homologs

In order to search characterized proteins sharing the identity with MdrP, we attempted to increase the number of maximum target sequences to 5,000, even 10,000 in the setting of algorithm parameters of BlastP at the NCBI website. However, the aligned protein sequences were still uncharacterized MFS transporters from a variety of microorganisms, even though the identity decreased to around 30%. Also, none of characterized or predicted Na^+^/H^+^ antiporters or proteins reported to exhibit Na^+^/H^+^ antiport activity were found to share any identity with MdrP. Since there are too many predicted proteins derived from the increasingly sequenced genomes in the Genbank database, it seems rather difficult to obtain the characterized proteins sharing the identity with MdrP by using BlastP at the NCBI website. Considering that TCDB is a specific database used for the collection of transmembrane transporters including many characterized and predicted MFS transporters (Saier et al., 2016), we further aligned MdrP using Blast at the TCDB website and found that it shares a quite low identity with the solely two characterized DHA1 family multidrug efflux pumps, *L. lactis* LmrP (28%, accession version No. CAA61918.1) and *E. coli* MdtH (22%, accession version No. P69367.1) (Supplementary Figure S2). However, alignment of MdrP with those two multidrug efflux pumps showed that Motif A with the consensus sequence of GxLaDrxGrkxxl exists between TMS2 and TMS3 of MdrP (Supplementary Figure S2), which is the signature motif of DHA family drug efflux pumps within MFS (Henderson and Maiden, 1987; Griffith et al., 1992; Paulsen et al., 1996).

### Growth Tests for Salt Tolerance and Alkaline pH Resistance

Because Na^+^/H^+^ antiporters extrude Na^+^ or Li^+^ to the exterior of cells by the influx of H^+^ (Krulwich et al., 2011; Quinn et al., 2012; Meng et al., 2014; Padan, 2014), this category of transporters can exhibit not only the tolerance to salts such as Na^+^ or Li^+^ but also offer the resistance to alkaline pH in the presence of Na^+^ or Li^+^. For the identification of the exact ORF with Na^+/^H^+^ antiport activity, *mdrP* gene was constructed at the priority into an expression vector pET19b through the fusion of the sole ORF of MdrP in frame with an N-terminal His_6_ tag (Supplementary Figure S1) and the resultant construct was designated pET-mdrP (Supplementary Table S1). To rule out the possibility that 5′-end truncated *uvrD* gene may be functional in *E. coli*, it was also subcloned similarly to the strategy of subcloning *mdrP* and the resultant construct was designated pET-truncated uvrD (Supplementary Table S1). Sequencing analysis revealed that MdrP or N-terminus truncated UvrD succeeded in being fused in frame with an N-terminal His_6_ tag. Either subclone was tested by functional complementation of its transformant with *E. coli* KNabc. As shown in **Figure 1A**, all *E. coli* KNabc and its transformants showed the normal growth in the absence of NaCl or LiCl. In contrast, *E. coli* KNabc/pET-MdrP, as well as KNabc/pUC-PM29, could grow in the presence of 0.2 M NaCl or 5 mM LiCl whereas KNabc/pET-truncated uvrD, as well as the negative controls KNabc/pET19b or KNabc/pUC18, showed no growth under the same stress conditions (**Figure 1A**). More detailed growth tests were carried out by using *E. coli* KNabc/pET-mdrP and KNabc/pET19b for the analysis on salt tolerance and alkaline pH resistance by MdrP. The results confirmed that KNabc/pET-mdrP could grow well in the presence of 0.2 M NaCl (**Figure 1B**) or 5 mL LiCl (**Figure 1C**). However, KNabc/pET19b was not able to grow under the same stress conditions (**Figures 1B,C**). Also, the expression of *mdrP* gene could offer *E. coli* KNabc the resistance to alkaline pH at 8.0 in the presence of 50 mM NaCl, at which the growth of KNabc/pET19b was completely inhibited (**Figure 1D**). Therefore, MdrP is exactly likely to function as a Na^+^/H^+^ antiporter.

### Topological Analysis of MdrP and Its Localization in the Cytoplasmic Membranes

**FIGURE 2:**
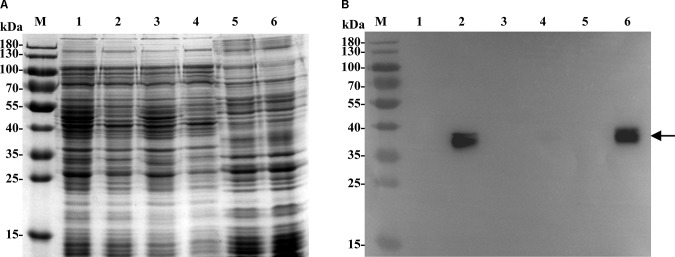
Localization of MdrP by western blot in the cytoplasmic membranes of *E. coli* KNabc. For the establishment of MdrP being a transmembrane protein, the samples for membrane fraction, cytoplasmic fraction and cell extract were prepared from cells of *E. coli* KNabc/pET19b (Lanes 1, 3, 5) and KNabc/pET-mdrP (Lanes 2, 4, 6), followed by the analyses of SDS-PAGE **(A)** and western blot **(B)**. The position of the target protein MdrP fused with an N-terminal His_6_ tag shown with a solid arrow.

Since all Na^+^/H^+^ antiporters are transmembrane proteins (Krulwich et al., 2011; Quinn et al., 2012; Meng et al., 2014; Padan, 2014), transmembrane segment prediction and hydropathy analysis were performed at the TCDB website to check whether MdrP is a transmembrane protein. As shown in Supplementary Figure S2, MdrP is predicted to be a transmembrane transporter of low polarity with 12 hydrophobic transmembrane segments (TMSs) including TMS 1 (18–37), TMS 2 (43–62), TMS 3 (74–94), TMS 4 (102–127), TMS 5 (137–161), TMS 6 (167–186), TMS 7 (225–250), TMS 8 (276–295), TMS 9 (305–324), TMS 10 (328–348), TMS 11 (372–391) and TMS 12 (395–414). For the establishment of MdrP being a transmembrane protein, the samples for cell extract, cytoplasmic fraction and membrane fraction were prepared from cells of *E. coli* KNabc/pET-mdrP and KNabc/pET19b, followed by the detection and localization of MdrP by western blot. The expression of MdrP was detected in cell extract and membrane fraction from *E. coli* KNabc/pET-mdrP but not in those from KNabc/pET19b (**Figure 2**). However, there was no positive signal in cytoplasmic fractions of *E. coli* KNabc/pET-mdrP or KNabc/pET19b (**Figure 2**). It should be pointed out that positive signals with two bands were detected, which may suggest that MdrP exists as an MFS transporter in the reduced and oxidized forms as reported by Jiang D. et al. (2013). On the basis of the above results, MdrP should be located in the cytoplasmic membranes of *E. coli* KNabc.

### Na^+^(Li^+^, K^+^)/H^+^ Antiport Assays

**FIGURE 3:**
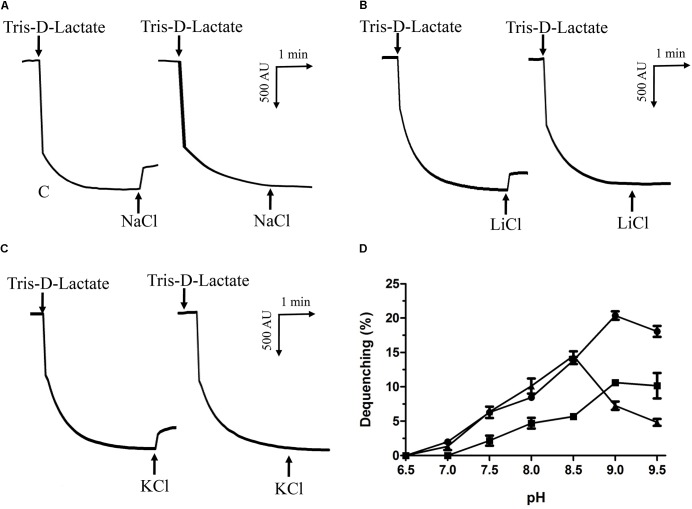
Na^+^(Li^+^, K^+^)/H^+^ antiport activity by MdrP and its activity pH profile. Na^+^(Li^+^, K^+^)/H^+^ antiport activity was determined in everted membrane vesicles prepared from cells of *E. coli* KNabc/pET-mdrP (to the left) or KNabc/pET19b (to the right) by the French pressure cell method. The optimal antiport activity at pH 9.0 for Na^+^/H^+^ **(A)** and Li^+^/H^+^ **(B)**, and at pH 8.5 for K^+^/H^+^ **(C)** were shown as the representatives of each of them. At the time points indicated by downward arrows, Tris-D-lactic acid (final concentration at 5 mM) was added to the assay mixture to initiate fluorescence quenching. At the time points indicated by upward arrows, NaCl (final concentration at 5 mM), LiCl (final concentration at 5 mM) or Na-free KCl (final concentration at 5 mM) was added to the assay mixture, respectively. Fluorescence quenching is shown in arbitrary units (AU). The antiport activity pH profile **(D)** for Na^+^/H^+^ (filled circle), Li^+^/H^+^ (filled square) and K^+^/H^+^ (filled triangle) were also plotted at the indicated pH values. The wavelength of excitation light was 492 nm and fluorescence was monitored at 526 nm. Each value point represents the average of three independent determinations.

**FIGURE 4:**
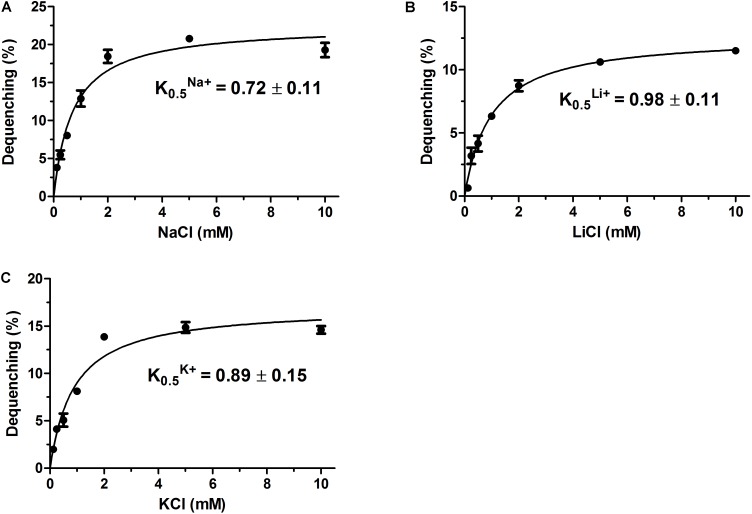
Calculation of *K*_0.5_ values of MdrP for Na^+^, Li^+^, and K^+^. The optimal antiport activity was determined at pH 9.0 for Na^+^/H^+^ **(A)** and Li^+^/H^+^ **(B)**, and at pH 8.5 for K^+^/H^+^ **(C)** at the varied cation concentrations from 0 to 10 mM. *K*_0.5_ values of MdrP for the tested cations were calculated on the basis of non-linear regression analysis with the software Prism 5.0 by the plotting of the antiport activity as the respective functions of the corresponding cation concentrations. Each value point represents the average of three independent determinations.

Na^+^/H^+^ antiporters were reported to simultaneously exhibit Na^+^/H^+^ and Li^+^/H^+^ antiport activity, and also sometimes K^+^/H^+^ antiport activity (Krulwich et al., 2011; Quinn et al., 2012; Meng et al., 2014; Padan, 2014). Therefore, everted membrane vesicles were prepared from *E. coli* KNabc carrying pET-mdrP or the negative control pET19b to confirm whether MdrP exactly functions as a Na^+^(Li^+^)/H^+^ antiporter or also as a K^+^/H^+^ antiporter. A fluorescence dequenching method with the acridine orange as a pH indicator was employed to measure Na^+^(Li^+^, K^+^)/H^+^ antiport activity by using everted membrane vesicles. The results showed that MdrP possesses not only Na^+^/H^+^ (**Figure 3A**) and Li^+^/H^+^ (**Figure 3B**) antiport activity but also K^+^/H^+^ (**Figure 3C**) antiport activity. Also, Na^+^/H^+^ and K^+^/H^+^ antiport activity was detected within a range of pH 7.0–9.5 whereas Li^+^/H^+^ antiport activity was detected within a range of pH 7.5–9.5 (**Figure 3D**). The optimal antiport activity was at pH 9.0 for Na^+^/H^+^ and Li^+^/H^+^ but at pH 8.5 for K^+^/H^+^ (**Figure 3D**). As a representative of cation concentration corresponding to half-maximum antiport activity, *K*_0.5_ values of MdrP for Na^+^, K^+^, and Li^+^ can be used for the evaluation of the apparent affinity of MdrP for the cations. *K*_0.5_ values of MdrP for Na^+^, Li^+^ and K^+^ were calculated to be 0.72 ± 0.11 mM (**Figure 4A**), 0.98 ± 0.11 mM (**Figure 4B**) and 0.89 ± 0.15 mM (**Figure 4C**), respectively. This reveals that MdrP should transport the cations with the preference of Na^+^ > K^+^ > Li^+^.

### Phylogenetic Analysis for MdrP

**FIGURE 5:**
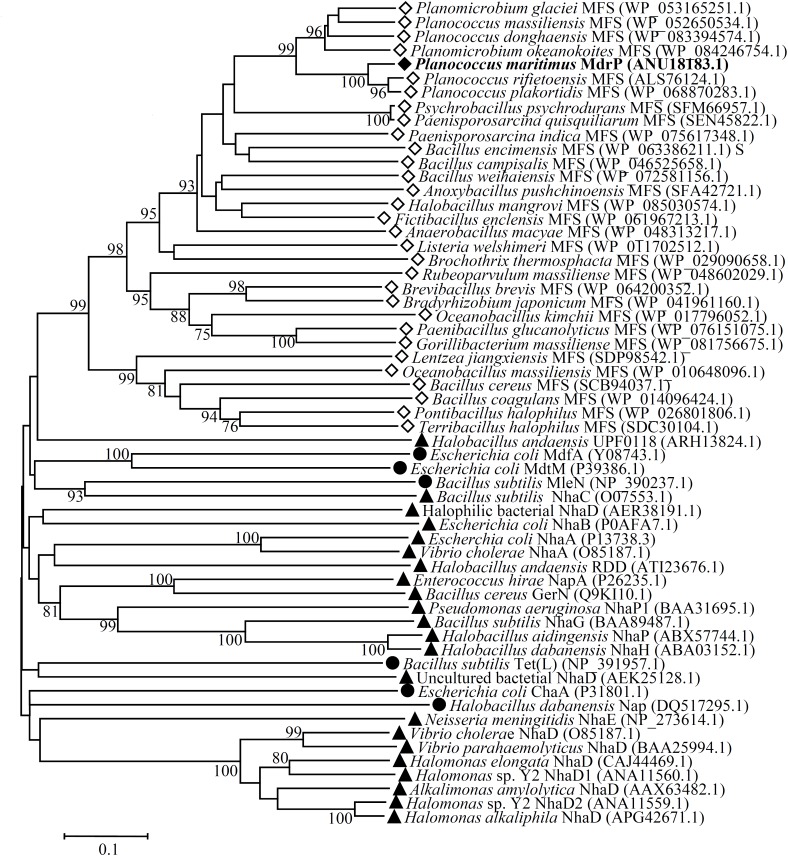
Neighbor-joining phylogenetic tree of MdrP and its selected homologs, together with single-gene proteins with Na^+^/H^+^ antiport activity. For the construction of this phylogenetic tree, ten closest homologs with 60–95% identities, ten closer homologs with 40–59% identities and ten distant homologs with 29–39% identities, together with all characterized single-gene proteins with Na^+^/H^+^antiport activity were selected. Accession version numbers of selected homologs were shown in the parenthesis. Filled diamond stands for MdrP, open diamond stands for putative MFS homologs, filled triangle stands for known single-gene Na^+^/H^+^ antiporters, filled circle stands for other single-gene proteins with Na^+^/H^+^antiport activity. Bootstrap values ≥ 70% (based on 1000 replications) were shown at branch points. Bar, 0.1 substitutions per amino acid residue position.

Since MdrP functions as a Na^+^(Li^+^, K^+^)/H^+^ antiporter but it shares no identity with all characterized or predicted Na^+^/H^+^ antiporters or proteins reported to exhibit Na^+^/H^+^ antiport activity, we speculate that MdrP may represent a novel class of Na^+^/H^+^ antiporters. To establish this speculation, we constructed the phylogenetic tree of MdrP with its putative MFS homologs plus all characterized single-gene proteins with Na^+^/H^+^ antiport activity. To guarantee the representativeness of MFS homologs, we selected ten closest homologs with 60–95% identities, ten closer homologs with 40–59% identities and ten distant homologs with 29–39% identities on the basis of the alignment of MdrP by using BlastP at the NCBI website. The criteria for the selection of homologs is that the identity with MdrP is at least 1–2% and/or homologs are from different microorganisms as possible. As shown in **Figure 5**, MdrP clustered with all its MFS homologs with the bootstrap value of 100%, which was significantly distant with all characterized single-gene proteins with Na^+^/H^+^ antiport activity including MdfA, MdtM, and Tet(L).

**FIGURE 6:**
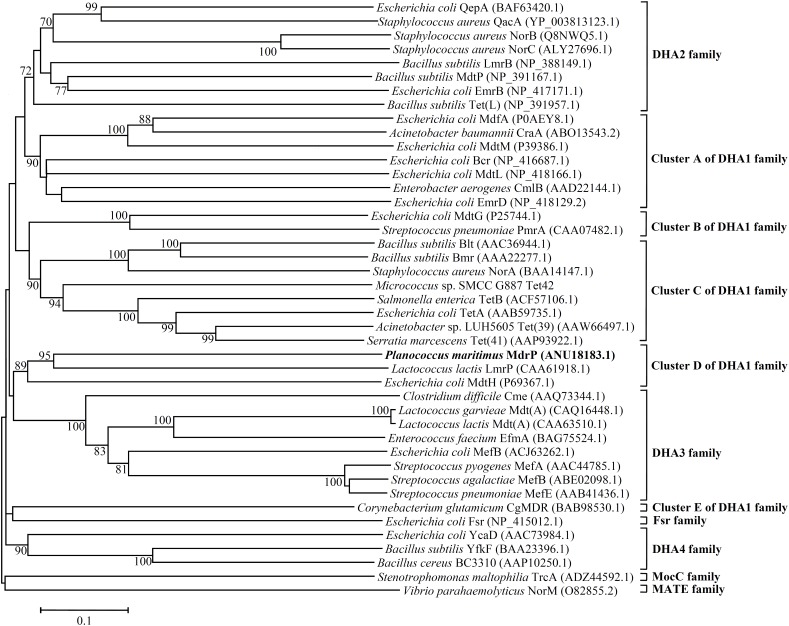
Neighbour-joining phylogenetic tree of MdrP and the MFS multidrug efflux pumps. For the construction of this phylogenetic tree, the representatives of characterized DHA1-3 members, the solely characterized DHA4 member BC3310 and two predicted members of this family, YcaD and YfkF, and all other family of characterized MFS drug efflux pumps were selected. A MATE family multidrug efflux pump NorM was used as an outgroup. Accession version numbers of selected homologs were shown in the parenthesis. Bootstrap values ≥ 70% (based on 1000 replications) were shown at branch points. Bar, 0.1 substitutions per amino acid residue position.

Since three MFS drug efflux pumps including MdfA (Edgar and Bibi, 1997), MdtM (Holdsworth and Law, 2013), and Tet(L) (Cheng et al., 1994) have been reported to exhibit Na^+^/H^+^ antiport activity, it seems to be possible that MdrP may function as a drug efflux pump. Although MdrP shares a quite low identity with LmrP and MdtH, MdrP was found to exactly contain the signature Motif A of DHA family drug efflux pumps within MFS (Supplementary Figure S2). Therefore, we further attempted to construct the phylogenetic tree of MdrP with MFS drug efflux pumps. To find out the possible drug resistance by MdrP based on the phylogenetic relationship, we selected the representatives of characterized DHA1-3 members, the solely characterized DHA4 member BC3310 and two predicted members of this family, YcaD and YfkF, and all other families of characterized drug efflux pumps within MFS plus a MATE family multidrug efflux pump NorM (Morita et al., 1998) as an outgroup. DHA2-4 family members constituted stable separate clusters with the bootstrap values above 70%, respectively (**Figure 6**). Also, one member of either Fsr family or MocC family showed a quite distant relationship with DHA family members as expected (**Figure 6**). These results established the reliability of phylogenetic analysis. As the largest DHA family within MFS, DHA1 family members constituted five stable separate clusters (Clusters A–E) with the bootstrap values above 70%, respectively (**Figure 6**). Of these five clusters, MdrP indeed clustered (Cluster D) solely with LmrP and MdtH with a bootstrap value of 89%. This suggests that MdrP may function as a multidrug efflux pump just like LmrP, MdtH or both.

### Resistance of MdrP to Antimicrobial Drugs

To test the resistance of MdrP to antimicrobial drugs, we constructed a drug-sensitive *E. coli* mutant CM2 through the disruption of one major multidrug efflux system, AcrAB, of *E. coli* DH5α. MICs of antimicrobial drugs were tested at the priority by using *E. coli* CM2 carrying pET-mdrP together with wild-type *E. coli* DH5α as a positive control, and CM2 with and without pET19b as two negative controls. As expected, MdrP showed the resistance to a number of antimicrobial drugs including ethidium bromide, clarithromycin and azithromycin that can be extruded by LmrP, and norifloxacin that can be extruded by MdtH, and other antibiotics such as gentamicin, kanamycin, and spectinomycin, but not erythromycin, tetracycline or rifampicin (**Table 1**).

**Table 1:** Minimal inhibition concentrations (MICs) of the tested antimicrobial drugs for wild-type *Escherichia coli* DH5α and its drug-sensitive mutant CM2 with no plasmid, pET19b or pET-mdrP.

Drug	Minimum inhibitory concentrations (μg/ml)			
	DH5α	CM2 only	CM2/pET19b	CM2/pET-mdrP
Ethidium bromide	200	100	100	200
Norifloxacin	0.2	0.025	0.025	0.05
Clarithromycin	128	16	16	32
Azithromycin	16	8	8	16
Gentamicin	4	4	4	16
Kanamycin	6.25	6.25	6.25	12.5
Spectinomycin	40	20	20	40
Erythromycin	400	50	50	50
Tetracycline	3.13	0.39	0.39	0.39
Rifampicin	12.5	6.25	6.25	6.25

### Determination of MdrP as a Multidrug Efflux Pump

**FIGURE 7:**
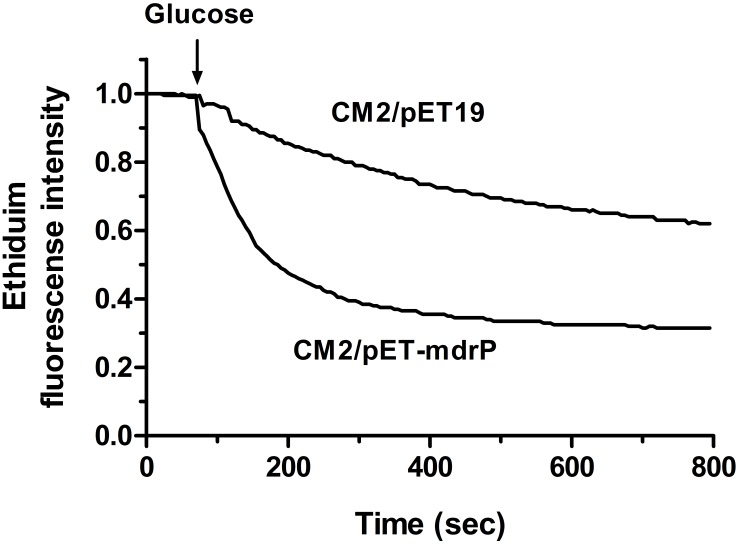
Assay for ethidium efflux by whole cells of *E. coli* CM2 transformants. Ethidium efflux assay was carried out by using the whole cells of *E. coli* CM2 transformants carrying pET-mdrP and the empty vector pET19b (as a negative control). CCCP was used at a final concentration of 40 μM for the depletion of cell energy and energy-starved cells were pre-loaded with ethidium bromide at a final concentration of 2.5 μM. After 1 min (downward arrow), glucose was added to cell suspensions at a final concentration of 20 mM to re-energize cells. The efflux of intracellular ethidium was monitored continuously by measuring the fluorescence of ethidium at the excitation and emission wavelengths of 500 and 580 nm, respectively.

**FIGURE 8:**
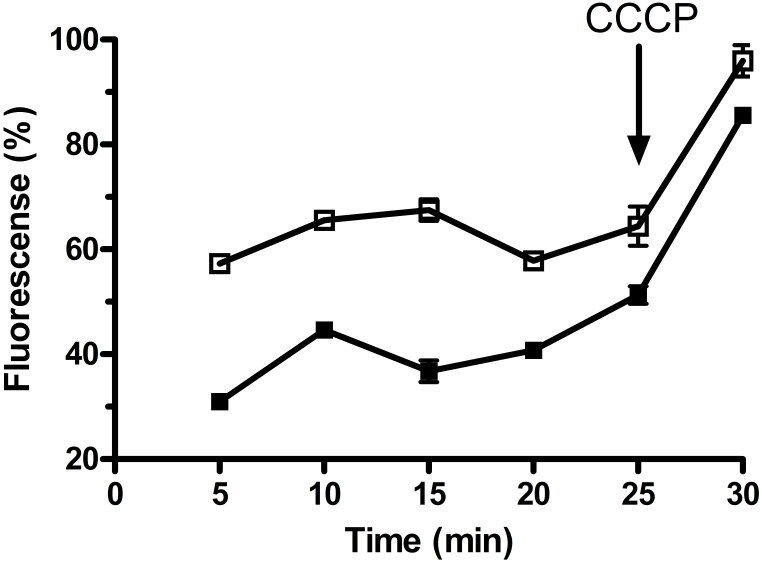
Assay for norfloxacin accumulation in whole cells of *E. coli* CM2 transformants. Norfloxacin accumulation was measured in the whole cells of *E. coli* CM2 transformants carrying pET-mdrP (filled square) and the empty vector pET19b as a negative control (open square). Norfloxacin was used at a final concentration of 100 μM to pre-load the cells. After 25 min, CCCP was added to the cell suspensions at a final concentration of 100 μM for the disruption of transmembrane proton gradient. Each portion of cells were sampled on the indicated time points, and the intracellular accumulated norfloxacin extracted from the cells was determined by measuring the fluorescence of norfloxacin at the excitation and emission wavelengths of 277 and 448 nm, respectively. Each value point represents the average of three independent determinations.

To analyze the function of MdrP as a multidrug efflux pump, we selected two representative antimicrobial drugs, ethidium bromide and norifloxacin, to test whether the whole cells expressing *mdrP* gene can display the efflux activities of these two drugs. For the measurement of ethidium efflux by MdrP, the energy-starved whole cells prepared from *E. coli* CM2/pET-mdrP and CM2/pET19b as a negative control were preloaded with ethidium bromide, followed by the addition of glucose to energize the cells. As shown in **Figure 7**, rapid ethidium efflux was observed with the cells of CM2/pET-mdrP just after the addition of glucose. In contrast, a slow efflux of ethidium was observed with the cells of CM2/pET19b. Norfloxacin efflux activity by MdrP was determined by comparing the difference of norfloxacin accumulation in the whole cells between *E. coli* CM2/pET-mdrP and CM2/pET19b. After the whole cells were preloaded with the same concentration of norfloxacin, a significantly lower level of norfloxacin accumulation was observed in the cells of CM2/pET-mdrP as the incubation time increased, as compared with those of CM2/pET19b (**Figure 8**). Also, norfloxacin accumulation could be dramatically increased to almost the same level in the whole cells of *E. coli* CM2/pET-mdrP and those of CM2/pET19b after the addition of CCCP, indicating that norfloxacin efflux by MdrP should be energy-dependent (**Figure 8**).

## Discussion

Numerous bacterial genomes have been increasingly sequenced and released at a relatively low cost due to the developed sequencing techniques. The annotation of protein function has been carried out mainly based on the sequence alignment, which leads to the failure of a considerable number of proteins in the prediction of their functions. As the largest and most diverse known superfamily of secondary transporters (Pao et al., 1998; Law et al., 2008; Reddy et al., 2012), MFS includes 27 functionally uncharacterized UMF families and many uncharacterized members categorized into well-characterized families due to their sharing no or significantly low sequence identity with the characterized MFS members (Saier et al., 2016). In the genome of *P. maritimus*DSM 17275^T^, 28 MFS transporters without the experimentally functional analysis have been non-annotated before this study. Here we present the characterization of one of them, MdrP, with the accession version No. ANU18183.1 from this strain. On the basis of protein alignment and phylogenetic analysis, we propose that MdrP should represent a novel class of Na^+^(Li^+^, K^+^)/H^+^ antiporters. More importantly, we found that this novel MFS transporter could also function as a multidrug efflux pump belonging to DHA1 family with a broad spectrum of drug resistance.

Bacterial Na^+^/H^+^ antiporters are a category of secondary transmembrane transporters that extrude Na^+^ and Li^+^ and sometimes also K^+^ in exchange for external H^+^, which play crucial roles in the maintenance of toxic monovalent cations at an acceptable cytoplasmic level and Na^+^/K^+^-dependent intracellular pH homeostasis under alkaline conditions (Krulwich et al., 2011; Quinn et al., 2012; Meng et al., 2014; Padan, 2014). Known Na^+^/H^+^ antiporters can be classified on the basis of the number of encoding genes into three major groups: (i) single-gene Na^+^/H^+^ antiporters including NhaA and NhaB (Herz et al., 2003), NhaC (Ito et al., 1997), NhaD (Zhang et al., 2014; Wang et al., 2017; Yang et al., 2018), NheE (Sousa et al., 2013), NhaG (Gouda et al., 2001), NhaH (Jiang et al., 2013b), NhaP (Utsugi et al., 1998), NapA (Waser et al., 1992), and GerN (Southworth et al., 2001); (ii) double-gene Na^+^/H^+^ antiporters including PsmrAB (Jiang et al., 2013a) and UmpAB (Meng et al., 2017); and (iii) multiple-gene Na^+^/H^+^antiporters such as Mrp (Cheng et al., 2016; Xu et al., 2018), Mnh (Hiramatsu et al., 1998), Pha (Jiang et al., 2004; Yang et al., 2006a) or Sha (Kosono et al., 1999). In addition, three MFS multi-drug efflux pumps such as MdfA (Edgar and Bibi, 1997), MdtM (Holdsworth and Law, 2013) and Tet(L) (Cheng et al., 1994), and a HCT (2-hydroxy-carboxylate transporter) family transporter MleN (Wei et al., 2000), and a primary Na^+^ pump Nap of NDH (NADH dehydrogenase) family (Yang et al., 2006b); and an UPF0118 family protein (Dong et al., 2017), and a RDD family protein (Shao et al., 2018) have also been increasingly reported to be able to function as Na^+^/H^+^ antiporters. In this study, MdrP was predicted to be a transmembrane protein with 12 putative TMSs (Supplementary Figure S2), which was established by the localization of MdrP by western blot in the cytoplasmic membranes of *E. coli* KNabc (**Figure 2**). Growth tests (**Figure 1**) and Na^+^(Li^+^, K^+^)/H^+^antiport assay (**Figure 3**) reveal that MdrP functions as both a Na^+^ (Li^+^)/H^+^ antiporter and a K^+^/H^+^ antiporter. However, protein alignment using BlastP (Altschul et al., 1990) at the NCBI website and phylogenetic analysis (**Figure 5**) reveals that MdrP is significantly different from the above-mentioned known Na^+^/H^+^ antiporters or proteins with Na^+^/H^+^ antiport activity. Therefore, we propose that MdrP should represent a novel class of Na^+^(Li^+^, K^+^)/H^+^ antiporters.

Most bacteria were predicted to contain 5–9 distinct single-gene or multiple-gene Na^+^/H^+^antiporters (Krulwich et al., 2009; Mesbah et al., 2009). However, we speculate that halophiles may have evolved significantly more Na^+^/H^+^ antiporters including even unreported ones to exhibit high capability of halo-alkaline tolerance, which has been demonstrated by our recent reports on several novel Na^+^/H^+^ antiporters from the different moderate halophiles (Dong et al., 2017; Meng et al., 2017; Shao et al., 2018). In these studies, we found that the members of three functionally uncharacterized families such as UPF0118, DUF1538 and RDD display Na^+^(Li^+^)/H^+^ or Na^+^(Li^+^, K^+^)/H^+^ antiport activity (Dong et al., 2017; Meng et al., 2017; Shao et al., 2018). Although a random screening method by functional complementation with *E. coli* KNabc helped us find out their functions as Na^+^/H^+^ antiporters, we suspect that these ever uncharacterized proteins may possess the additional functions, whose function predictions are, to great extent, restricted by limited bioinformatic knowledge about these uncharacterized proteins with no or significantly low sequence identity with their homologs. However, a similar dilemma to MdrP may be overcome, since MFS members have been reported to transport a diverse range of substrates such as drugs or cations, and etc. (Law et al., 2008; Reddy et al., 2012). Enlightened by the reports of three MFS members, MdfA (Edgar and Bibi, 1997), MdtM (Holdsworth and Law, 2013), and Tet(L) (Cheng et al., 1994), with Na^+^/H^+^ or Na^+^(K^+^)/H^+^ antiport activity, we speculate that MdrP may also function as a drug efflux pump, as well as a Na^+^(Li^+^, K^+^)/H^+^ antiporter. As a representative of a novel class of Na^+^/H^+^ antiporters, MdrP showed quite a distant phylogenetic relationship with the above-mentioned drug efflux pumps with Na^+^/H^+^ antiport activity (**Figure 5**). Also, MdrP shares a quite low identity with LmrP and MdtH (Supplementary Figure S2). However, we found that Motif A exactly exists between TMS2 and TMS3 of MdrP (Supplementary Figure S2), which is the signature motif of DHA family drug efflux pumps within MFS (Henderson and Maiden, 1987; Griffith et al., 1992; Paulsen et al., 1996). This suggests that MdrP is likely to function as amultidrug efflux pump. More importantly, phylogenetic analysis between MdrP and the MFS drug efflux pumps were introduced to analyze the correlation of MdrP with its possibly transported drugs. Among all characterized MFS drug efflux pumps, MdrP indeed constitute a separate cluster solely with LmrP and MdtH with a bootstrap value of 89% (**Figure 6**), further supporting the speculation that MdrP may function as a multidrug efflux pump just like LmrP, MdtH or both. That was established by the results of MIC tests for antimicrobial drugs (**Table 1**) and assays for drug efflux pump activity (**Figures 7**, **8**). Therefore, MdrP should exactly function as amultidrug efflux pump.

We are so interested in why MdrP, as well as MdfA (Edgar and Bibi, 1997), MdtM (Holdsworth and Law, 2013), and Tet(L) (Cheng et al., 1994), can simultaneously exhibit drug efflux activity and Na^+^/H^+^antiport activity. Two multidrug efflux pumps of MATE family designated NorM from *Vibrio parahaemolyticus* and *Neisseria gonorrhoeae* were reported to display drug efflux activity, which was stimulated by the presence of Na^+^(Li^+^) or Na^+^(K^+^), and proposed that they should function as Na^+^/multidrug antiporters (Morita et al., 2000; Long et al., 2008). Drug efflux by NorM from *V. cholerae* was also established to be able to be driven by either sodium motive force or proton motive one (Jin et al., 2014). In this study, Norfloxacin accumulation by MdrP was guaranteed to be measured in the absence of Na^+^, Li^+^, or K^+^ and found to be disrupted by the addition of CCCP (**Figure 7**), indicating that norfloxacin efflux by MdrP may be energy-dependent. CCCP as a H^+^ ionophore can be used for the disruption of pH gradient across the cytoplasmic membranes. However, when proton motive force is strongly decreased by the addition of CCCP, sodium motive force will be also disappeared. Therefore, whether norfloxacin efflux activity by MdrP can also be stimulated by the presence of Na^+^, Li^+^, or K^+^ remains to be identified in the future study.

Taken together, the results presented in this study provide a strong evidence that an uncharacterized MFS transporter belonging to DHA1 family exhibits dual functions as a Na^+^ (Li^+^, K^+^)/H^+^antiporter and a multidrug efflux pump. This will be very helpful to not only positively contribute to the function prediction of uncharacterized MFS members especially DHA1 family ones, but also broaden the knowledge of Na^+^/H^+^ antiporters. Also, the finding of this novel MFS transporter may provide a good basis for the further exploration about why DHA1 family drug efflux pumps also can exhibit Na^+^/H^+^antiport activity and how drug efflux pumps especially Na^+^-dependent ones share Na^+^-or-drug-transporting channel with Na^+^/H^+^ antiporters.

## Author Contributions

JJ and HA-M were responsible for the design of this study. HA-M, LM, ZZ, AA, LS, TX, FM, SA, and RZ carried out the experiments. JJ and HA-M analyzed the data and prepared figures and tables. HA, LM, ZZ, AA, and LS interpreted the results of experiments. HA-M drafted the manuscript. JJ edited and revised the manuscript. All authors discussed and approved the final version.

## Conflict of Interest Statement

The authors declare that the research was conducted in the absence of any commercial or financial relationships that could be construed as a potential conflict of interest.
