# Supplementary material for: An Uncharacterized Major Facilitator Superfamily Transporter From Planococcus maritimus Exhibits Dual Functions as a Na+(Li+, K+)/H+ Antiporter and a Multidrug Efflux Pump
Source: Front Microbiol. 2018 Jul 16;9:1601. doi: 10.3389/fmicb.2018.01601 (PMC6055358; doi:10.3389/fmicb.2018.01601)
Supplement: Supplementary file 1 [file Table_1.PDF]

frontiers

Supplementary Material

An uncharacterized major facilitator superfamily transporter from *Planococcus maritimus* exhibits dual functions as a $Na^+(Li^+, K^+)/H^+$ antiporter and a multidrug efflux pump

Heba Abdel-motaal, Lin Meng, Zhenglai Zhang, Amro Abdelazez, Shao Li, Tong Xu, Fankui Meng, Shaima Abozaed, Rui Zhang, Juquan Jiang[*]

*** Correspondence:** Juquan Jiang: jjqdainty@163.com

Table S1. Strains, plasmids and primers used in the study.

Strains, plasmids or primers	Relevant phenotype, genotype or primer sequence	The source or usage
Strains		
Planococcus maritmus DSM17275^T	Type strain of *Planococcus maritmus*, a slightly halophile	Ordered from German Collection of Microorganisms and Cell Cultures (DSMZ)
Escherichia coli KNabc	*nhaA*::Km^R, *nhaB*::Em^R, *chaA*::Cm^R	Donated by Dr. Terry A. Krulwich
Escherichia coli DH5α	F$^-$, φ80d*lacZ* ΔM15, Δ(*lacZYA-argF*)U169, *deoR* , *recA1*, *endA1*, *hsdR17*(*rK*$^-$, *mK*$^+$), *phoA*, *supE44*, *λ*$^-$, *thi*$^{-1}$, *yrA96*, *relA1*	Takara Biotechnology (Dalian) Co., Ltd., China
Escherichia coli CM2	A major multidrug efflux system-deficient *E. coli* DH5α mutant (Δ*acrAB*), which was obtained by homologous recombination	This study
Plasmids		
pUC18	Cloning vector	Takara Biotechnology (Dalian) Co., Ltd., China
pUC-PM29	pUC18 carrying a 3.2 kb DNA fragment including 5'-end truncated *uvrD* and *mdrP*	This study
pET19b	Over-expression vector	Novagen Ltd., USA
pET-mdrP	pET19 carrying *mdrP* gene	This study
pET-truncated uvrD	pET19 carrying 5'-end truncated *uvrD*	This study
pKD46	λ Red recombinase expression plasmid with ampicillin resistance	Donated by Dr. W. Todd Lowther
pKD3	Template plasmid for a chloramphenicol resistance gene	Donated by Dr. W. Todd Lowther
Primers		
mdrP_F	5'-CATATGAAGATCAAGGATTGGAA-3' (*Nde*I, undelined）	For the subcloning of *mdrP* gene
mdrP_R	5'-GGATCCTTATATGTTCGCAACCGTT-3' (*Bam*HI, underlined)	
uvrD_F	5'-CATATGCTCGAGGAATTGGTCTCATT-3' (*Nde*I, undelined）	For the subcloning of 5'-end truncated *uvrD* gene
uvrD_R	5'-GGATCCTTATATCAGTTCCAGCAATT-3' (*Bam*HI, underlined)	
acrAB_KF	5'-ATGAACAAAAACAGAGGGTTTACGCCTCTGGCGGTCCATATGAATATCCTCCTTA-3' (*Eco*RI, underlined)	For the knockout of *acrAB* genes
acrAB_KR	5'-TCAATGATGATCGACAGTATGGCTGTGCTCGATATCTGTGTAGGCTGGAGCTGCTTC-3’(*Eco*RI, underlined)	
acrAB_VF	5'-TTAGGAAGGAAGCGGGACA-3'	For the verification of *acrAB* knockout
acrAB_VR	5'-AGTGGATCGCCAGGGAATT-3'	

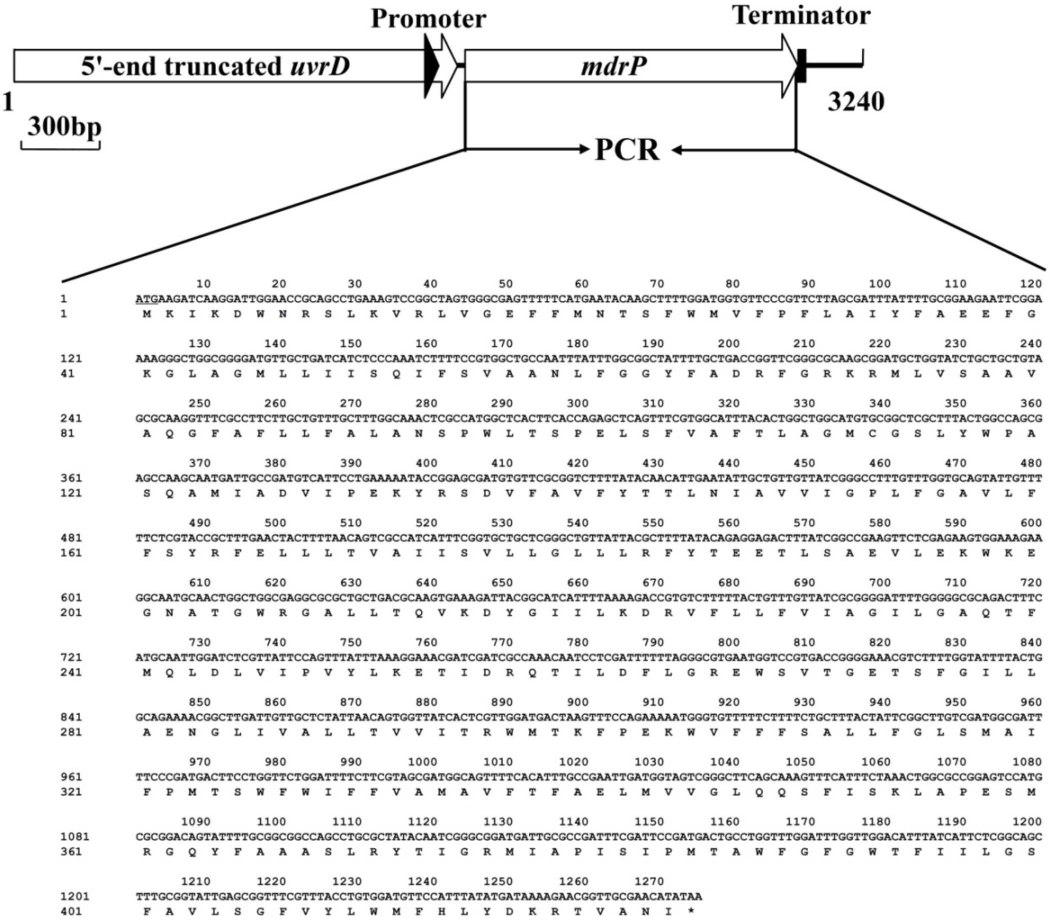

Fig. S1. Mapping of the inserted DNA fragment in the recombinant plasmid pUC-PM29 and subcloning of *mdrP* gene.

One 5'-end truncated *uvrD* and one intact *mdrP* are included in this 3.2-kb DNA fragment. The former begins from No. 1 bp of this DNA fragment whereas the latter is preceded by respective promoter-like sequence (filled arrow) and a respective Shine-Dalgarno (SD) sequence (filled rectangle). The sequence of *mdrP* gene from its initiation codon (ATG, underlined) to stop codon (TAA, marked with the star) was subcloned by PCR amplification and fused in frame with an N-terminal His_6 tag into an expression vector pET19b.

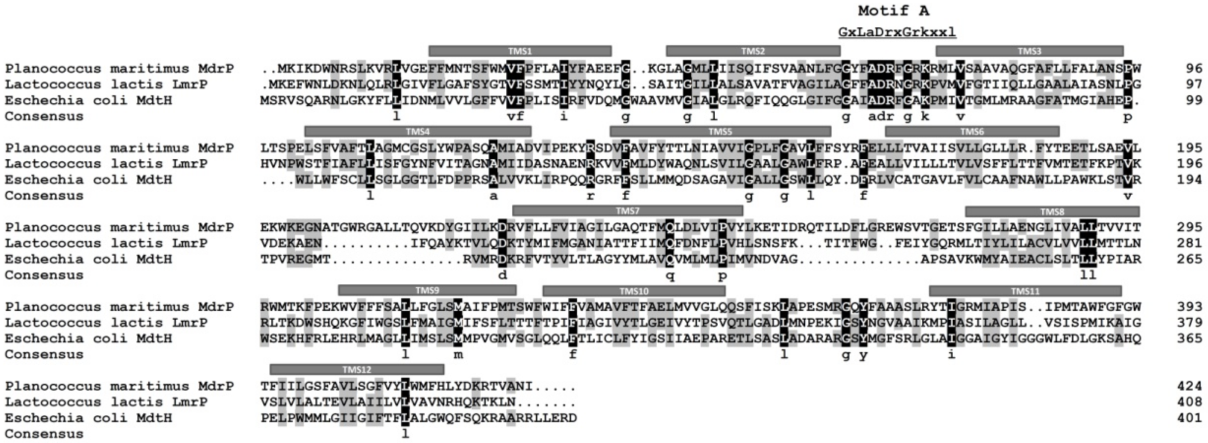

Fig. S2. Alignment of MdrP with the solely two characterized DHA1 family multidrug efflux pumps.

MdrP was aligned with the solely two characterized DHA1 family multidrug efflux pumps, *Lactococcus lactis* LmrP (28 %, accession version No. CAA61918.1) and *Escherichia coli* MdtH (22 %, accession version No. P69367.1). Shading homology corresponds to 100 % (black), ≥ 50 % (grey) and < 50 % (white) amino acid identity, respectively. The twelve putative transmembrane segments (TMSs) are marked with grey bars above the alignment. The consensus sequence of Motif A was highlighted above the alignment between TMS2 and TMS3.
